# A Review of Recent Advances in Conversion and Self-Assembled Anti-Corrosion Films for Copper and Its Alloys

**DOI:** 10.3390/molecules31162869

**Published:** 2026-08-17

**Authors:** Kangwei Gongsun, Xiang Gao, Changfeng Zhao, Houyi Ma

**Affiliations:** 1 Key Laboratory of Colloid and Interface Chemistry of State Education Ministry, School of Chemistry and Chemical Engineering, Shandong University, Jinan 250100, China; 202112181@mail.sdu.edu.cn; 2 Engineering & Technology Center of Electrochemistry, School of Chemistry and Chemical Engineering, Qilu University of Technology (Shandong Academy of Sciences), Jinan 250353, China; 3 School of Energy and Mechanical Engineering, Dezhou University, Dezhou 253023, China; zhaochf16@dzu.edu.cn

**Keywords:** copper, conversion coatings, covalent bonding, self-assembled monolayers (SAMs), multifunctional coatings

## Abstract

Copper and its alloys are indispensable for electronics, communications, new energy systems, and aerospace engineering due to their exceptional electrical conductivity and mechanical properties. However, the thin cuprous oxide (Cu_2_O) layer that naturally forms on copper and its alloys is prone to failure under elevated temperatures and high humidity, particularly in chloride-rich environments, leading to accelerated localized corrosion. While conventional chromate-based passivation has long been the industrial standard for preventing corrosion, its use has been increasingly restricted by global regulations (such as RoHS and REACH) due to its severe toxicity and health risks. To address the conflict between environmental compliance and protective performance, this review systematically evaluates recent advances in environmentally friendly, chromium-free anti-corrosion coatings in the present review. These alternative coatings are critically analyzed and categorized into four mechanistic groups: (i) inorganic conversion coatings (including molybdate, tungstate, rare earth, and phosphate systems); (ii) organic films formed via chemical or physical adsorption (such as organic inhibitors, thiol-based monolayers, and organosilane self-assembled films); (iii) conversion coatings engineered through covalent bonding, coordination chemistry, and microstructural tailoring; and (iv) multifunctional coatings that integrate self-healing capability with high electrical conductivity. Beyond providing a technical summary, this review explored how the swift progression of electronic information technology, new energy infrastructure, and robotics has imposed more exacting, multifunctional demands on copper components. This review provides a strategic roadmap for future research and prioritizes the creation of protection strategies that operate robustly in multi-physics coupling environments—integrating high conductivity, autonomous self-healing, and long-term chemical stability to ensure the reliability of next-generation infrastructure.

## 1. Introduction

### 1.1. Introduction

Copper possesses unparalleled advantages across a wide range of industrial applications: its electrical and thermal conductivities are among the highest of all non-precious metals, making it a key material for efficient electrical energy transmission and thermal management, while its exceptional ductility and malleability facilitate precise fabrication, ranging from micron-scale wires to complex structural components. Through alloying, the strength, wear resistance, and other functional properties of copper can be flexibly tailored. The resulting alloys can form self-protective oxide films in diverse environments, exhibiting outstanding corrosion resistance and long-term service stability [1]. The unique synergy of high-efficiency electrical and thermal conduction, structural and functional integration, environmental tolerance, and ecological friendliness has rendered this material indispensable across power electronics, high-end manufacturing, green energy, and emerging technologies [2]. In strategic emerging industries such as information electronics, robotics, and aerospace, the application of copper has evolved from conventional macroscopic components to microscopic, mission-critical elements that govern system-level performance limits. Additionally, the global transition toward electric vehicles has significantly intensified the performance requirements for copper components used in battery systems and power electronics [3,4,5]. These transitions have subjected copper to increasingly severe operational environments: nanoscale interconnects in integrated circuits face electromigration risks; thermal end-effectors on spacecraft endure extreme temperatures and atomic oxygen erosion; and high-dynamic robot cables experience coupled cyclic mechanical stress and corrosive degradation [6,7,8]. Emerging application scenarios demand that copper materials possess high strength, high stability, and the ability to withstand extreme environments. Consequently, corrosion and oxidation—age-old material failure mechanisms—have reemerged in more complex forms, becoming the core bottlenecks restricting technological breakthroughs and reliability in these cutting-edge fields [9,10].

Traditional chromate passivation has historically been the predominant surface treatment for protecting copper and copper alloys against corrosion, oxidation, and discoloration [11,12,13]. This process entails immersing the copper substrate in an acidic solution containing hexavalent chromium (Cr(VI)), in forms such as chromic acid or potassium dichromate, whereby redox reactions induce the formation of a non-crystalline composite passivation layer, typically from 10 to several hundred nanometers thick. The passivation layer consists predominantly of chromium(III) oxide (Cr_2_O_3_) and a minor amount of copper oxide, providing dual corrosion protection through both a physical barrier and anodic inhibition. However, owing to its well-established environmental persistence and severe human health risks, including carcinogenicity, mutagenicity, and ecotoxicity, chromate-based passivation has been progressively phased out by the global manufacturing industry. Regulatory initiatives, such as the European Union’s Restriction of Hazardous Substances (RoHS) Directive and the Registration, Evaluation, Authorisation and Restriction of Chemicals (REACH) Regulation, explicitly prohibit or severely restrict hexavalent chromium compounds, driving high-end sectors, including electronics, automotive, and consumer appliance manufacturing, to adopt safer, environmentally compliant alternatives [14].

**Figure 1:**
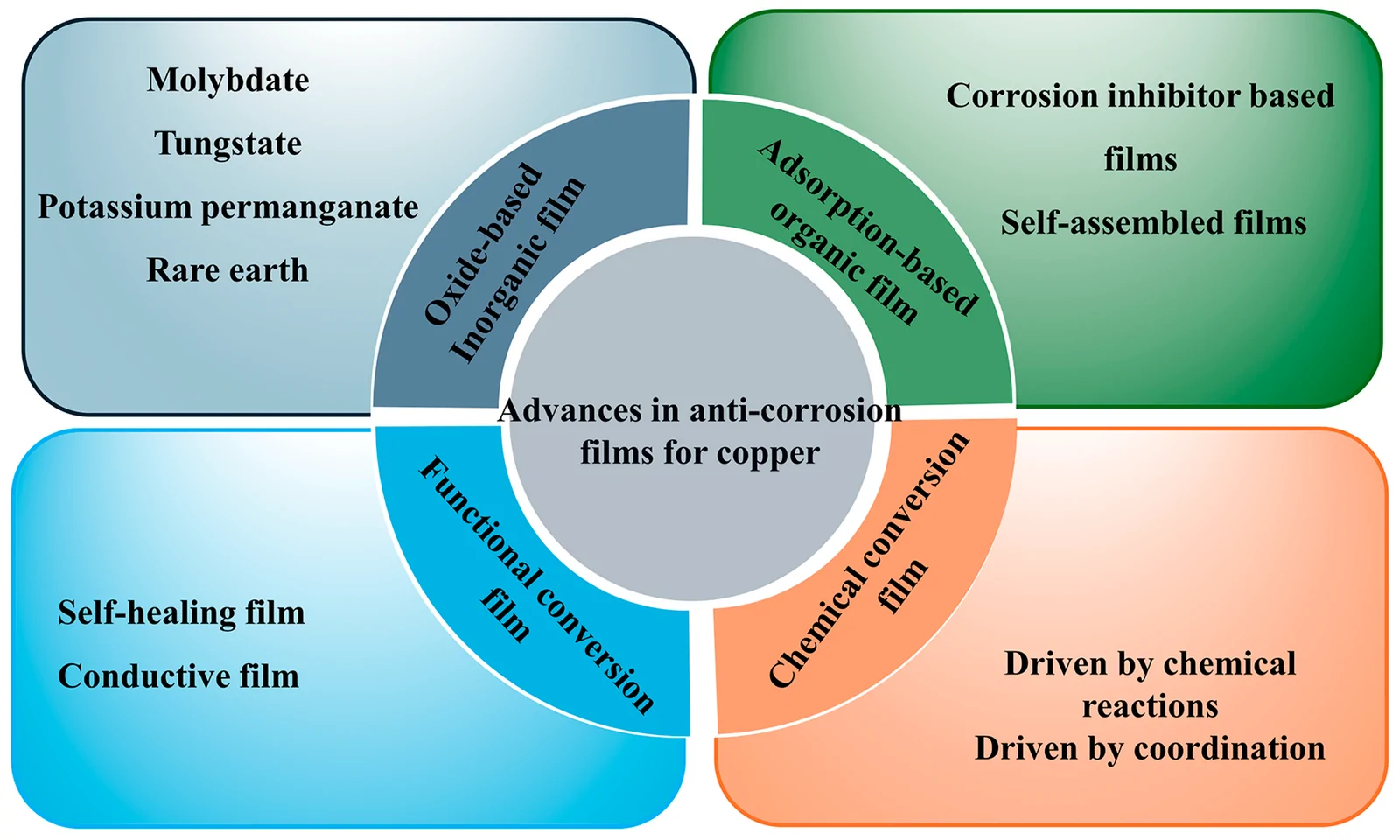
Recent advances in anti-corrosion films for copper.

In response to the environmental and regulatory limitations of conventional chromate passivation, recent research has focused on developing environmentally benign, high-performance, and multifunctional non-chromate conversion coatings for copper and copper alloys. As illustrated in Figure 1, these approaches can be categorized as follows: (i) inorganic oxide-based films, designed to emulate the protective functionality of Cr(VI) passivation while eliminating chromium entirely; (ii) specific adsorption-driven organic films, formed via chemisorption or physisorption of corrosion inhibitors (e.g., benzotriazole (BTA)) or self-assembled monolayers (SAMs) (e.g., alkanethiols and organosilanes); (iii) chemical conversion films: substances bearing active functional groups are anchored to the copper substrate through specific reactions between the modifier molecules and the substrate, after which a dense protective thin film is constructed on the copper surface via interactions including chemical bonding, coordination, covalent bonding, and other intermolecular forces; and (iv) engineered multifunctional coatings that integrate corrosion resistance, oxidation resistance, and electrical conductivity, and some of which incorporate self-healing capabilities, to meet the stringent demands of emerging applications in electronics, electric vehicles, and advanced packaging. To enhance coating performance, strategies involving controlled film thickening and interfacial engineering, including approaches such as optimizing the substrate/coating adhesion and interfacial chemistry, have been systematically pursued. In this review, we critically summarize recent advances in eco-friendly conversion coatings for copper-based substrates, identify persistent technical challenges, including long-term stability under operational conditions, scalability of deposition processes, and standardized performance evaluation, and outline promising directions for next-generation surface protection technologies.

### 1.2. Literature Search Methodology

To ensure a comprehensive and systematic overview of the field, a structured literature search was conducted. Primary academic databases, including Web of Science and Google Scholar, were utilized to identify relevant studies. The search strategy employed combinations of the following keywords: (copper or copper alloys or Cu) and (corrosion or corrosion protection or anti-corrosion or coating) and (conversion coating or self-assembled monolayer or SAMs or hybrid film or film or passive).

The publication period was primarily 2000 to 2025, with particular emphasis on recent advances from 2018 onwards to capture state-of-the-art developments. The inclusion criteria were defined as follows: (i) studies focusing specifically on copper or its alloys; (ii) research involving environmentally friendly (chromium-free) surface treatments; and (iii) papers providing quantitative performance metrics (e.g., inhibition efficiency or salt spray hours) and mechanistic insights. Studies involving traditional chromates were included only for comparative purposes. An initial pool of approximately 850 records was identified. After screening titles and abstracts for relevance and removing duplicates, around 150 full-text articles were critically assessed, and 80 representative studies were ultimately selected for detailed discussion in this review.

## 2. Corrosion and Oxidation Mechanisms of Copper and Associated Evaluation Methodologies

### 2.1. Multi-Scale Degradation Mechanisms

Copper corrosion is a complex electrochemical process heavily influenced by the presence of aggressive anions (particularly Cl^−^) and the pH–potential (E−pH) environment. In chloride-containing media, the anodic dissolution of copper does not proceed directly to stable oxides but involves the production of intermediate species. Initially, at lower anodic potentials, copper undergoes a one-electron oxidation to form cuprous ions (Cu^+^). In the presence of chloride ions, these ions rapidly react to form an insoluble adsorbed layer of cuprous chloride (CuCl_ads_) on the surface [15]:

Cu + Cl^−^ → CuCl_ads_ + e^−^ (1)

**Figure 2:**
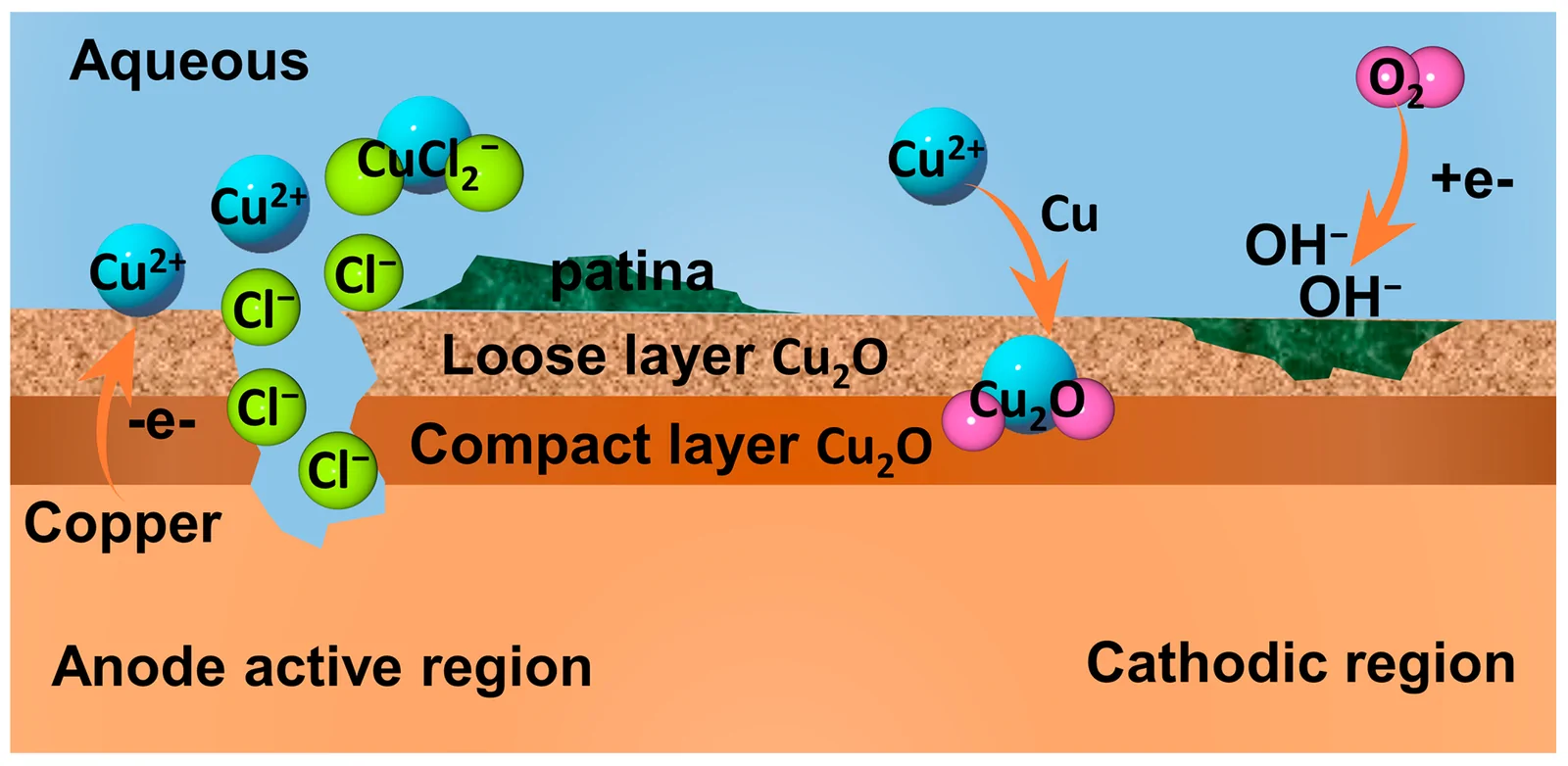
Diagram of electrochemical corrosion of copper in chlorine-containing solution.

As shown in Figure 2, in environments with high chloride concentrations, this CuCl film further reacts with excess Cl^−^ to form the highly soluble cuprous-dichloro complex (CuCl_2_^−^), which is the primary source of mass loss:

CuCl_ads_ + Cl^−^ → CuCl_2_^−^ (2)

The formation of protective oxides is highly pH-dependent. In neutral or slightly alkaline conditions, the hydrolysis of CuCl_2_^−^ or the direct oxidation of copper leads to the formation of a Cu_2_O layer:

2CuCl_2_^−^ + 2OH^−^ → Cu_2_O + H_2_O + 4Cl^−^ (3)

However, the stability of this passivating layer is compromised in acidic environments (pH < 5), where Cu_2_O becomes thermodynamically unstable and dissolves into Cu^2+^ ions. Furthermore, chloride ions can locally penetrate the Cu_2_O lattice via an anion-exchange mechanism or through physical defects, disrupting the film’s continuity and triggering localized pitting corrosion. This E−pH-dependent competition between the growth of passivating oxides (Cu_2_O/CuO) and the formation of soluble chloride complexes (CuCl_2_^−^) dictates the overall corrosion rate and the transition from uniform to localized attack [16,17,18]:

O_2_ + 2H_2_O + 4e^−^ → 4OH^−^ (neutral or alkaline conditions) (4)

or

O_2_ + 4H^+^ + 4e^−^ → 2H_2_O (acidic conditions) (5)

It should be noted that the initially formed cuprous oxide (Cu_2_O) is a p-type semiconductor with inherently limited passivating ability. Under more oxidizing conditions or in alkaline, CO_2_-rich, or SO_2_-containing environments, Cu_2_O may undergo further oxidation to copper(II) oxide (CuO) or react with atmospheric constituents to form secondary corrosion products, including basic copper carbonate (patina, Cu_2_(OH)_2_CO_3_) and basic copper sulfate (Cu_4_(OH)_6_SO_4_).

In the service environments encountered by copper-based materials, sulfide species, including H_2_S, HS^−^, and S^2−^, can react with copper to form highly conductive copper(I) sulfide (Cu_2_S), which enhances cathodic kinetics and promotes either rapid uniform corrosion or the development of non-adherent, porous sulfide scales [19].

### 2.2. Methodologies for Evaluating Corrosion and Oxidation Behavior of Copper-Based Alloys

**Figure 3:**
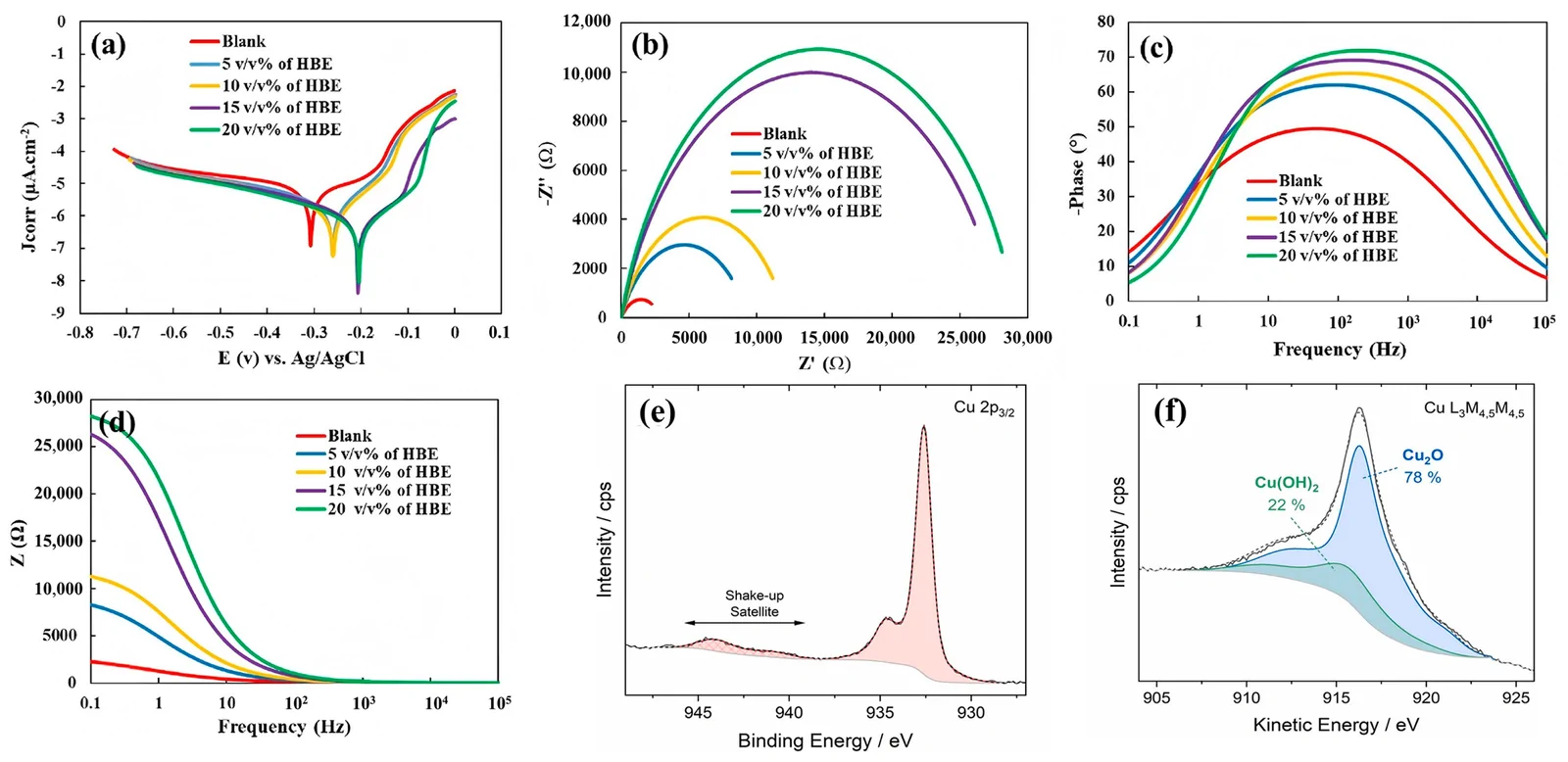
Corrosion assessment methods: (**a**) potentiodynamic polarization curve; (**b**–**d**) EIS [22]; (**e**) Cu 2p XPS high-resolution spectra; and (**f**) Cu LMM spectra for copper [24].

To comprehensively evaluate the corrosion resistance of copper and its alloys, an integrated assessment framework—spanning from rapid laboratory screening to service-representative environmental simulation—is essential. As illustrated in the schematic (Figure 3), these methodologies provide complementary data on the interfacial, structural, and chemical stability of the protective strategies. In electrochemical characterization, potentiodynamic polarization provides a quantitative assessment of corrosion tendency (*E*_corr_), the uniform corrosion rate (*i*_corr_), and pitting susceptibility. For copper alloys in halide-rich environments, the pitting breakdown potential (*E*_pit_) is a decisive parameter: a wider passive region and a more noble *E*_pit_ relative to *E*_corr_ signify superior resistance to the localized breakdown of the protective film. Simultaneously, Electrochemical Impedance Spectroscopy (EIS) enables the non-destructive, in situ characterization of interfacial processes. Through equivalent circuit modeling incorporating solution resistance (*R*_s_), charge transfer resistance (*R*_ct_), and constant-phase elements (CPE), EIS quantitatively evaluates film corrosion resistance. For copper, the diameter of the capacitive loop in the Nyquist plot serves as a direct marker for interfacial protection—a larger arc radius reflects a higher resistance to ionic transport. Furthermore, the inclusion of Warburg diffusion impedance is critical for modeling the diffusion-controlled kinetics typical of copper dissolution through its native Cu_2_O/CuO layers [20,21,22].

However, although potentiodynamic polarization and EIS are two core electrochemical techniques for investigating corrosion mechanisms, their corresponding data analysis strategies still have distinct limitations. The Tafel extrapolation method requires a well-defined Tafel region to ensure accurate measurement; when the electrode reaction is not controlled by activation polarization, the extrapolation results will be invalid. For EIS analysis, the most widely adopted approach is the equivalent circuit method, which simplifies complex electrochemical processes into combinations of passive components such as resistors and capacitors. Under specific conditions, different equivalent circuits can produce similar impedance response spectra, which may lead to substantial fitting deviations and erroneous interpretation of the electrode interface structure. Similar limitations are also present in fitting error evaluation, Kramers–Kronig validation, and the analysis of non-ideal electrochemical processes. Therefore, analyzing complex non-equilibrium corrosion processes by oversimplifying them into a small number of apparent parameters carries inherent methodological risks. The future development of corrosion electrochemistry should gradually shift from relying on averaged polarization curves and EIS to a new research paradigm that integrates microscale in situ characterization techniques with quantitative kinetic modeling, so as to extract intrinsic kinetic parameters with clear physical meaning.

In industrial service simulations, neutral salt spray (NSS) testing (e.g., ASTM B117-26) evaluates long-term resistance based on the time to first observable degradation (e.g., discoloration or pitting). Cyclic corrosion testing (CCT) incorporates controlled sequences of wetting, drying, and humidity cycling to more realistically replicate dynamic stresses, providing a more discriminating evaluation of real-world degradation than single-mode NSS. Additionally, humidity and heat testing are vital for electronic components and PCB assemblies, focusing on surface discoloration, coating blistering, and interfacial delamination under synergistic high-temperature and high-humidity conditions [23]. At the microscopic and chemical scale, SEM-EDS enables high-resolution imaging of corrosion morphology, pit geometry, and microcrack networks while mapping elemental distributions. To elucidate chemical states, X-ray photoelectron spectroscopy (XPS) provides a depth-resolved characterization of oxidation states. In copper research, while the Cu 2p spectrum identifies Cu^2+^ via its characteristic satellite peaks, the overlap between Cu(0) and Cu+ necessitates the use of Auger Electron Spectroscopy (AES). The Cu LMM kinetic energy shift and the calculated Auger parameter are indispensable for precisely differentiating metallic copper from cuprous oxide (Cu_2_O) [24]. Finally, X-ray diffraction (XRD) remains essential for macro-scale phase identification, allowing for the evaluation of the crystallinity and composition of complex corrosion products (e.g., malachite or hydroxychlorides), thereby confirming the long-term chemical stability delivered by the protective system. Among the Top Ten Scientific Questions in Electrochemistry, deeply elucidating the multi-step electrode reaction kinetics during corrosion processes is recognized as a critical research topic [25]. Atomic force microscopy (AFM) enables nanoscale, three-dimensional topographic imaging of material surfaces, with quantitative characterization of surface roughness parameters and localized electrochemical activity via scanning Kelvin probe force microscopy (SKPFM). This provides a technical means for the in-depth study of interfacial electrode reaction kinetics during corrosion processes [26,27,28]. To meet functional stability requirements in electrical and electronic applications, electrical resistivity and thermal diffusivity measurements have been integrated into the corrosion evaluation framework. The quantitative assessment of post-corrosion changes in these properties allows for a direct correlation between degradation severity and critical device performance criteria: current-carrying capacity, thermal management efficacy, and operational safety margins [29].

Nowadays, computational chemistry has become an indispensable tool for studying the atomic-scale mechanisms of copper protection. These theoretical approaches provide a “bottom-up” perspective on how molecular structures affect macroscopic inhibition performance. This multi-scale combination from macroscopic experiments to microscopic structures provides a comprehensive understanding of the interface between the copper substrate and the protective film, bridging the gap between molecular electronic structure and macroscopic barrier performance. First-principles calculations, based on Density Functional Theory (DFT), served as a rigorous method for quantifying the thermodynamic stability and reactive activation of the film/substrate interface [30]. Herein, the primary indicator of interface stability is the adsorption energy (*E*_ads_), which characterizes the binding strength between the corrosion inhibitor molecules and specific copper lattice plane (e.g., Cu(111), Cu(100)).

*E*_ads_ = *E*_total_ − (*E*_surf_ + *E*_mol_) (6)

**Figure 4:**
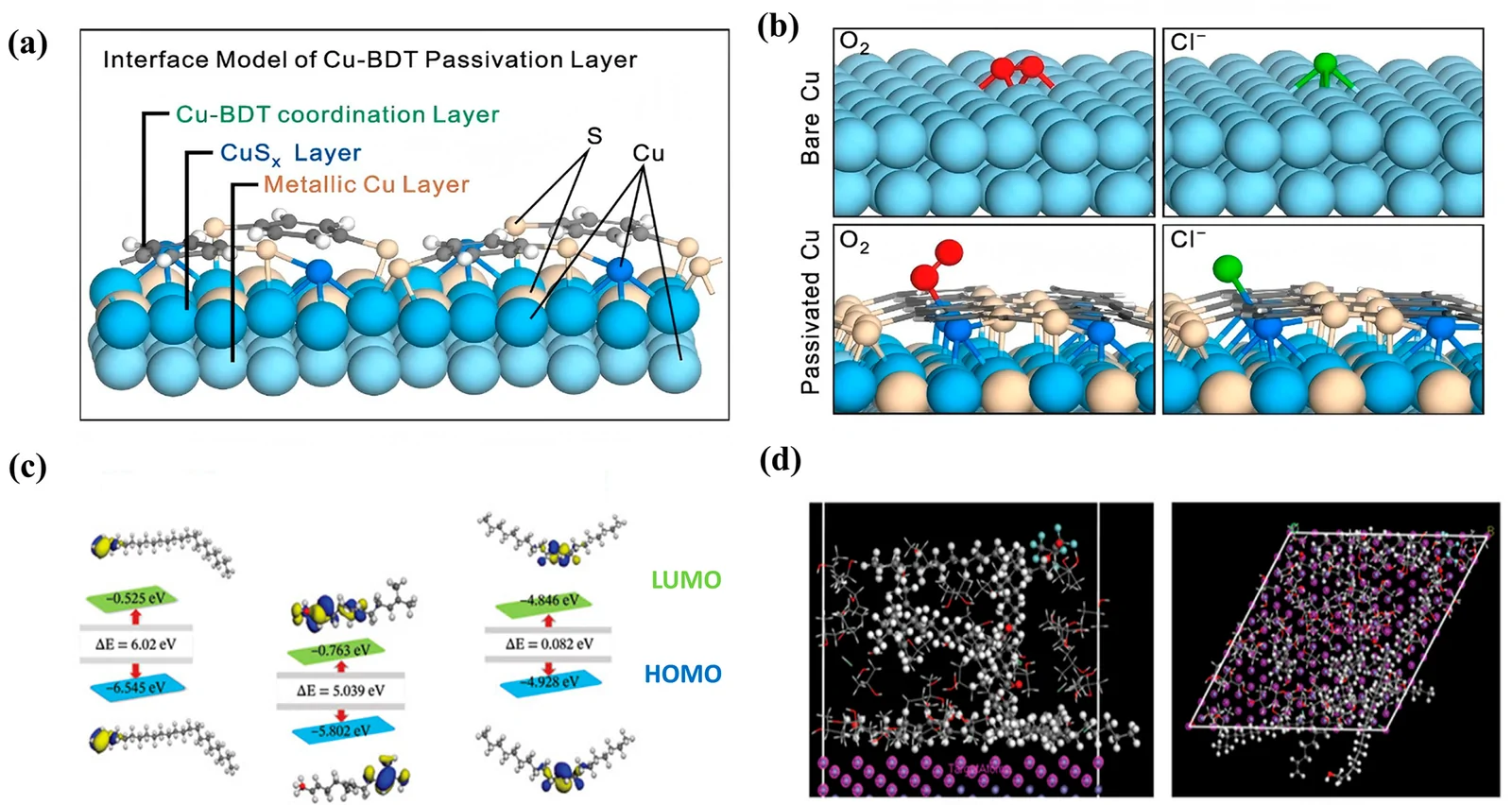
Adsorption of corrosion inhibitors (**a**) and corrosive species (**b**) on copper surface [31]; the HOMOs and LUMOs of corrosion inhibitors (**c**); the adsorption structure between corrosion inhibitors and metal (**d**) calculated through MD simulation [33].

A more negative *E*_ads_ signifies a more spontaneous adsorption process. She et al. utilized first-principles calculations to reveal the atomistic protective mechanism (Figure 4), confirming that the bilayer structure significantly elevates the adsorption energy barriers for corrosive species (such as O_2_ and Cl^−^) on the copper surface, thereby effectively inhibiting the corrosion process from a kinetic perspective [31]. To understand the chemical basis of this binding, frontier molecular orbital theory is employed. The energies of the highest occupied molecular orbital (HOMO) and the lowest unoccupied molecular orbital (LUMO) provide insights into the ability of molecules to donate electrons to or accept electrons from the empty d-orbitals of copper atoms, respectively. A small HOMO–LUMO energy gap (Δ*E*) typically suggests high chemical reactivity and a stronger tendency to form coordinate bonds with the copper surface. Furthermore, Molecular Electrostatic Potential (ESP) mapping is used to visualize the charge distribution across the molecule. By identifying regions of high and low electron density on the ESP surface, researchers can pinpoint electrophilic and nucleophilic sites, thereby predicting the most probable adsorption configurations (e.g., vertical vs. flat-lying) and the resulting surface coverage. Moreover, charge density difference analysis is commonly employed to visualize electron redistribution and accumulation at the inhibitor–copper interface, thereby providing direct evidence of charge transfer. Complementing this, partial density of states (PDOS) analysis is utilized to confirm chemical bonding by revealing the orbital hybridization between lone pairs of the inhibitor’s heteroatoms (e.g., S, N, O) and the d-orbitals of the copper surface atoms. These DFT-derived parameters provide a theoretical blueprint for the rational modification of molecular structures to maximize their surface affinity.

To evaluate long-term barrier properties, molecular dynamics (MD) and Monte Carlo (MC) simulations are utilized to simulate the dynamic behavior of films at a larger scale [32,33]. MD simulations are particularly effective in assessing the ordering and compactness of self-assembled monolayers (SAMs) or hybrid coatings. By analyzing the density profiles of corrosive species (H_2_O, Cl^−^) near the interface, researchers can quantify the ability of films to retard the penetration of aggressive ions. A lower diffusion coefficient within the coating matrix typically correlates with superior barrier efficiency. MC simulations help identify the equilibrium adsorption states by stochastically searching the configuration space, ensuring that the modeled interface reflects the most energetically stable state.

Recently, machine learning (ML) has emerged as a transformative paradigm for the high-throughput screening of synergistic corrosion inhibitors [34,35]. By training models on extensive datasets of molecular descriptors and electrochemical performance, ML can identify complex structure–activity relationships (QSAR) and optimize the formulation of hybrid films. The synergy between atomistic simulations and ML-guided design represents a shift from trial-and-error discovery toward a predictive framework for developing ultrastable, multifunctional copper protection technologies.

## 3. Research Progress on Environmentally Friendly Surface Treatment Technologies

### 3.1. Inorganic Non-Chromium Oxidation Films

The exceptional corrosion protection afforded by hexavalent chromium passivation stems primarily from its potent oxidizing capability and intrinsic self-healing behavior. Specifically, Cr(VI) species are reduced on the metal substrate to form Cr(III), which subsequently co-precipitates with dissolved metal cations to yield a dense, amorphous mixed-oxide gel layer [36]. Building on this mechanistic understanding, contemporary research on inorganic non-chromium conversion films centers on two complementary strategies. The first is a substitutional approach, utilizing elements from the same periodic group or exhibiting comparable redox activity, focusing on transition metals such as molybdenum (Mo) and tungsten (W), whose multiple oxidation states and potent oxidizing capability allow them to emulate the chromate-driven oxidative film formation process [37,38,39]. The second strategy relies on cathodic inhibition mediated by rare earth elements, particularly cerium (Ce) and lanthanum (La), which selectively precipitate as insoluble hydroxides or oxides at local cathodic regions, thereby suppressing the oxygen reduction reaction and retarding overall corrosion [40,41]. 

In efforts to emulate the chromate system’s oxidative film formation pathway, molybdate- and tungstate-based systems have attracted considerable research interest. The proposed film-forming mechanism proceeds as follows: under acidic or neutral conditions, Mo(VI) or W(VI) species, which act as mild oxidants, are partially reduced on the copper substrate, yielding a mixed amorphous film predominantly composed of lower-valent molybdenum or tungsten oxides/hydroxides (e.g., Mo(IV)/Mo(V) or W(IV)/W(V)) and concurrently incorporating Cu(I)/Cu(II) oxides. Despite their structural coherence, these conversion films commonly exhibit micro-porosity, resulting in a corrosion protection performance that remains substantially inferior to that of chromate-based counterparts. In contrast, potassium permanganate (KMnO_4_) has emerged as a more extensively investigated alternative, as its strong oxidizing capability enables the direct formation of a dark brown to black conversion layer comprising manganese dioxide (MnO_2_) and copper oxides on the copper surface. Crucially, the film’s optical appearance, thickness, and microstructural features, including its crystallinity and phase distribution, can be precisely tuned by controlling key process parameters such as solution pH, temperature, and KMnO_4_ concentration.

To mitigate the microcracking prevalent in single-component potassium permanganate conversion coatings, hybrid systems incorporating molybdate or phosphate species are widely adopted to improve film density and corrosion resistance. Rare earth-based conversion coatings constitute a distinct protective strategy rooted in cathodic inhibition. In aerated electrolytes, the oxygen reduction reaction at cathodic sites on the copper surface raises the local interfacial pH, prompting the precipitation of Ce^3+^ as insoluble Ce(OH)_3_. This intermediate is subsequently “in situ” oxidized to cerium dioxide (CeO_2_), forming a mixed Ce(III)/Ce(IV) oxide/hydroxide layer preferentially localized at cathodic regions. By impeding the cathodic half-reaction, this deposit disrupts the electrochemical corrosion cell and retards overall anodic dissolution. Although rare earth coatings offer exceptional environmental benignity, their practical deployment is constrained by slow film growth kinetics, inherently low coating thickness, and inadequate stability in acidic environments. Furthermore, the high cost of high-purity rare earth precursors and challenges in reproducible process control hinder large-scale industrial adoption. Critically, copper’s thermodynamic stability and low standard electrode potential render its surface refractory to oxidation by mild oxidants such as tungstate, molybdate, and trivalent rare earth cations, thereby diminishing the effectiveness of chromate-mimetic passivation. As a result, such approaches achieve markedly higher efficacy and broader applicability for aluminum alloys and carbon steel [42,43,44], whereas their performance enhancement for copper and its alloys remains marginal. To bridge this gap, organic corrosion inhibitors, most notably BTA and organosilanes, are routinely incorporated into multi-step treatment schemes. These molecules adsorb onto and penetrate the porous inorganic framework, effectively sealing micro-defects and yielding a functionally integrated inorganic–organic bilayer with superior barrier integrity and sustained corrosion protection. 

In summary, considerable progress has been achieved in the development of inorganic non-chromium oxidative conversion coatings as technically viable and environmentally friendly alternatives to hexavalent chromium passivation. Of these, molybdate-based systems, particularly when integrated with phosphate or organic inhibitors, represent the most technologically mature and industrially validated approach, attributable to their close mechanistic alignment with chromate-mediated oxidation and their robust corrosion resistance under service conditions. Rare earth-based coatings offer an environmentally friendly alternative, with nanostructured modifications, such as the incorporation of CeO_2_ nanoparticles into silicate or hybrid organic–inorganic matrices, demonstrating marked improvements in deposition rate, coating thickness, and long-term barrier performance. Potassium permanganate (KMnO_4_)-derived coatings, while less effective for general corrosion protection, provide distinctive functional benefits in applications demanding high-contrast decorative finishes or where process economics dictate stringent cost constraints. Compared to conventional chromate passivation, inorganic non-chromium films like molybdates and rare earth systems offer superior environmental profiles. However, their primary limitation remains the intrinsic porosity and microcracking formed during their formation process, which can provide pathways for Cl^−^ penetration. For example, while certain molybdate films achieve decent inhibition, their long-term salt spray resistance is often inferior to hybrid systems due to these micro-defects. Future research should focus on synergistic doping with organic inhibitors or nano-oxides to seal micro-pores and the development of low-temperature, rapid-growth mechanisms to enhance industrial throughput.

### 3.2. Organic Thin Films Fabricated via Chemical or Physical Adsorption

Precision corrosion protection technologies for copper and its alloys, especially in applications requiring exceptional functional stability such as electronic information systems and high-end instrumentation, are undergoing a paradigm shift—from conventional macroscopic coatings to molecular-level, rationally designed surface regulation. Corrosion-inhibiting films, typified by BTA [45,46,47,48], along with self-assembled monolayer (SAM) strategies based on alkylthiols [49,50,51,52] and organosilanes [53,54,55], provide highly efficient, spatially controlled, and functionally benign resistance against corrosion and oxidation. This performance stems from the rational assembly of nanoscale-ordered protective architectures directly on copper surfaces. As a result, these approaches represent leading research frontiers and critical enabling strategies in advanced metal surface protection.

Corrosion inhibitors form monolayer or multilayer protective films on metal surfaces via physical or chemical adsorption, thereby preventing direct interaction between the corrosive medium and the underlying metal substrate. BTA and its derivatives represent the most thoroughly studied and broadly deployed class of copper corrosion inhibitors. The triazole ring nitrogen atoms in BTA coordinate with surface-confined Cu(I) species to yield a dense, water-insoluble polymeric complex film, formally designated as [Cu(I)–BTA]_n_. This film is anchored predominantly through chemisorption, affording high interfacial stability and effectively suppressing both the anodic dissolution of copper and cathodic oxygen reduction. Nevertheless, conventional BTA-based films exhibit insufficient long-term durability, as they are prone to hydrolytic cleavage and thermally or electrochemically induced structural rearrangement. Furthermore, their capacity to inhibit localized corrosion, especially pitting and crevice corrosion, is fundamentally limited, and their protective performance deteriorates markedly in aggressive, chloride-rich electrolytes.

To overcome these inherent limitations, current research focuses on two principal strategies: The first is the rational molecular design of BTA derivatives—for example, incorporating hydrophobic alkyl chains to enhance film hydrophobicity and barrier functionality, thereby improving moisture resistance, or introducing multifunctional chelating groups to enable multidentate coordination with surface Cu(I) species, thereby increasing film cohesion, packing density, and interfacial adhesion. The second is synergistic hybrid inhibition, in which BTA is co-formulated with functionally orthogonal inhibitors to construct hierarchically organized, multimodal protective architectures that exploit cooperative interfacial stabilization mechanisms. As a representative example, Lin et al. [45] developed a phytic acid (PA)/BTA composite conversion coating on copper via a controlled immersion process. At an optimized BTA loading of 1.6 wt%, the resulting coating exhibited a maximum water contact angle of 137.5° and an exceptionally low corrosion current density of 1.610 × 10^−7^ A/cm^2^. Complementary spectroscopic analyses confirm hydrogen-bonded cross-linking between the PA-derived phosphate–chelate network and the BTA–Cu(I) coordination polymer, yielding a denser, more defect-resistant interface with markedly enhanced corrosion resistance. Geuli et al. [46] fabricated a BTA–trimethylsilyl silicate (TMS) composite film on copper nanoparticles, reducing the copper dissolution rate in 3.5 wt% NaCl solution by one order of magnitude. Liu et al. [47] developed a hybrid BTA–organosilane (JH-M902) passivation film, termed the BTA–Si system, on electrolytic copper foil through a precisely controlled chemical immersion process. Electrochemical characterization revealed that the BTA–Si-modified copper exhibited a corrosion current density of 1.115 × 10^−6^ A/cm^2^ and an inhibition efficiency of 99.56% in 3.5 wt% NaCl solution, markedly surpassing the performance of conventional passivation films. Solmaz et al. [48] prepared a B-pollen film on a copper surface, and their quantitative electrochemical analysis confirmed a protection efficiency of 98.45% under standardized 3.5 wt% NaCl testing conditions. It should be emphasized that the corrosion inhibition efficiencies reported across the various studies in this review were obtained using a standardized corrosive medium of 3.5 wt% NaCl solution. Although this consistent use of a simulated marine environment provides a relatively comparable baseline for evaluating the initial protective potential of different coating systems, a direct comparison of absolute values still requires caution, as the immersion duration prior to electrochemical measurements (varying from 1 h to several days) and the grade of the copper substrate (e.g., pure copper vs. specific brass/bronze alloys) can influence the reported metrics. While these high efficiencies indicate exceptional short-term barrier properties, they should be interpreted alongside long-term durability data, such as salt spray resistance, to fully determine their industrial reliability.

SAM technology facilitates the formation of ultrathin, highly ordered films, exclusively limited to a single molecular layer, via the spontaneous, vectorial adsorption of amphiphilic molecules onto atomically clean, low-index crystalline metal surfaces. As a foundational platform in molecular-scale interface science, SAMs provide deterministic control over interfacial structure, chemical composition, and functional behavior. Among molecular adsorbates, alkanethiols form the most structurally uniform and extensively investigated SAMs on copper and gold surfaces [49,50,51,52]. The thiol (–SH) headgroup undergoes dissociative chemisorption at surface Cu atoms, forming a robust, covalent S–Cu bond with well-defined coordination geometry. Intermolecular van der Waals interactions between adjacent alkyl chains drive dense, two-dimensional molecular packing, enforcing an all-trans conformation and a well-defined tilt angle (~30° from the substrate normal), thereby yielding a long-range, crystalline-like monolayer architecture. The chemical identity of the terminal functional group governs the interfacial properties of the SAM, including surface wettability, chemical reactivity, electron-transfer kinetics, and corrosion inhibition capability. Flores et al. [49] fabricated dodecanethiol-based SAMs on copper substrates via immersion in an ethanol solution for 20 h under ambient conditions, and systematically evaluated their corrosion inhibition performance in 3.5 wt% NaCl electrolyte. Their study demonstrated that the SAMs function as an effective molecular-scale barrier, significantly suppressing both anodic copper dissolution and cathodic oxygen reduction reactions. Rao et al. [50] prepared alkylthiol-based SAMs on polycrystalline copper substrates and further evaluated their corrosion protection performance under ambient atmospheric exposure, revealing that the SAMs extend the copper substrate’s induction period for visible oxidation by up to 10 days. Nevertheless, thiol-based SAMs exhibit limited thermal stability and insufficient long-term chemical robustness under ambient oxidative and humid conditions. Under elevated temperatures (>80 °C) or in strongly oxidizing (e.g., H_2_O_2_-containing) or alkaline (pH > 10) environments, the S–Cu bond is susceptible to oxidative cleavage and interfacial debonding. Moreover, the formation of high-quality thiol-based SAMs imposes stringent requirements on substrate surface cleanliness, necessitating atomically clean, oxide-free copper surfaces achieved through rigorous pre-treatment protocols.

Organosilanes constitute another important class of SAM precursor, especially effective for modifying metal surfaces bearing a native oxide layer [52,53,54,55]. Following hydrolysis, the resulting silanol species condense with surface-bound hydroxyl groups on copper, forming robust Si–O–Cu covalent linkages. Concurrently, silanol molecules undergo intermolecular condensation to form a lateral Si–O–Si network, thereby establishing a three-dimensional polymeric architecture. Masmoudi et al. [53] investigated the hydrolysis kinetics of γ-aminopropyltriethoxysilane (γ-APS) in ethanol–water mixtures and subsequently deposited a γ-APS-derived self-assembled film onto copper substrates. The effects of hydrolysis duration and thermal curing time on film morphology, thickness, and interfacial stability were systematically evaluated. Gladkikh et al. [54] co-assembled organosilane-functionalized thiols with BTA, carboxylic acids, or phosphonic acids to construct a polymer-like protective film on copper substrates. EIS and accelerated atmospheric corrosion testing confirmed the film’s effectiveness in inhibiting copper oxidation under ambient conditions. While the organosilane-derived film architecture demonstrated superior interfacial stability relative to thiol-based self-assembled monolayers, the film formation involved sequential hydrolysis and condensation reactions, rendering process control highly sensitive to ambient moisture and solution pH. Moreover, uncontrolled condensation can promote heterogeneous multilayer formation or colloidal aggregation, thereby compromising both the thickness uniformity and interfacial continuity of the protective film. Organic SAMs, particularly alkanethiols and BTA-based films, provide high-precision protection for microelectronics while having a minimal impact on electrical and thermal conductivity. Nevertheless, they are highly susceptible to thermal failure and oxidative degradation during high-temperature processing, and the desorption of non-covalently bonded inhibitors remains a concern for long-term reliability. Research should move toward using multidentate anchoring molecules to increase grafting density and explore the thermal–oxidative stability of SAMs under extreme operational loads.

### 3.3. Conversion Coatings

Currently, research on the use of SAMs for copper surfaces primarily centers on thiols, nitrogen-containing heterocycles (e.g., benzotriazole), and organosilanes. The fabrication of these films typically relies on organic solvents, thereby elevating environmental hazards, increasing production costs, and impeding large-scale industrial adoption.

Furthermore, film-forming agents exhibit a largely unidimensional functionality: their active functional groups serve primarily to anchor molecules onto the substrate surface, without providing driving force for progressive, layer-by-layer assembly. Consequently, the resulting films are predominantly monolayers or non-covalently assembled multilayers, exhibiting limited structural robustness and insufficient long-term corrosion resistance. Therefore, the development of environmentally benign film systems capable of delivering sustained corrosion resistance is critically important.

In recent years, researchers have successfully developed diverse coordination-based assembly films in aqueous media for active metals, including magnesium, iron, and aluminum, demonstrating effective corrosion inhibition. However, copper’s inherently higher electrochemical nobility impedes the direct adaptation of these aqueous coordination systems. Moreover, achieving covalent surface assembly under aqueous conditions remains technically challenging due to competitive hydrolysis and limited interfacial reactivity. Therefore, advancing research on copper-specific conversion films with exceptional corrosion resistance holds dual significance: it addresses the longstanding bottleneck in fabricating robust surface films on copper substrates, while also offering transferable design principles for eco-friendly assembly coatings on other technologically relevant metals.

#### 3.3.1. Construction of Conversion Coatings Driven by Chemical Reactions

Conventional SAMs typically exhibit thicknesses ranging from several to tens of nanometers, inherently constraining further enhancement of their anti-corrosion performance. To overcome this limitation, engineering a sustained driving force for film formation—thereby enabling controlled, three-dimensional growth of SAMs—constitutes a viable strategy for enhancing anti-corrosion performance. Thiol groups demonstrate a high affinity for copper surfaces, undergoing spontaneous chemisorption to form robust S–Cu bonds with bond dissociation energies exceeding 200 kJ/mol.

**Figure 5:**
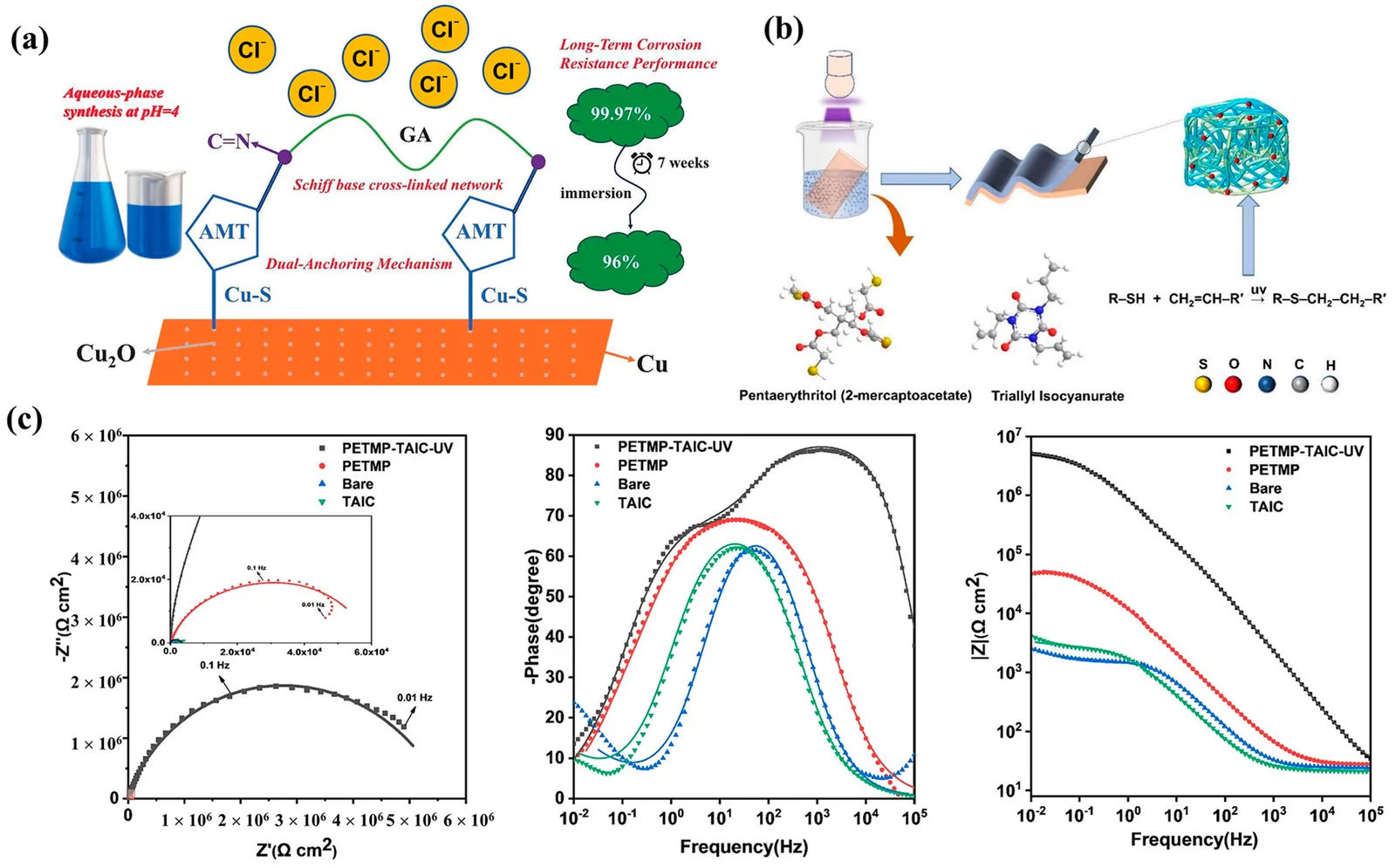
Fabrication of conversion coatings through Schiff base reaction (**a**) [59], Ultraviolet light initiation click reaction (**b**), and the EIS results of CuAAC-derived film (**c**) [61].

As illustrated in Figure 5, multifunctional thiol-containing molecules were selected as the primary building blocks for film formation. Through chemisorption mediated by thiol groups, these molecules are covalently anchored onto the copper substrate surface. Subsequently, complementary reagents capable of reacting selectively with the pendant functional groups exposed on the anchored monolayer are introduced into the film-forming solution. By precisely tuning key reaction parameters (e.g., temperature, concentration, and reaction time), the anchored molecules undergo iterative coupling via click chemistry and Schiff base condensation [56,57,58,59,60,61,62,63], enabling controlled, three-dimensional growth of the monolayer. This approach enables the fabrication of monolayer-derived films with precisely tunable thickness and exceptional anti-corrosion performance. Qiu et al. [56] employed 3,6-dioxa-1,8-octanedithiol (DOT), a bifunctional dithiol, as a self-assembly precursor. By leveraging copper–thiol chemisorption for initial surface anchoring, followed by click coupling between DOT’s terminal thiol groups and epoxy or vinyl ester functionalities in silane coupling agents, they achieved hierarchical, micrometer-thick surface architectures on copper. EIS and potentiodynamic polarization measurements revealed protection efficiencies of 99.6% and 99.4%, respectively. Mechanistic investigations revealed that DOT molecules first undergo chemisorption onto the copper surface via Cu–S bond formation; subsequently, the distal thiol groups react with epoxy or vinyl ester functionalities of the silane coupling agents to form stable C–S linkages. This sequential, orthogonal coupling process initiates the in situ construction of a covalently cross-linked network, thereby enabling sustained, three-dimensional film growth. Zhang et al. [57] employed pentaerythritol tetrakis(2-mercaptoacetate) (PETMP) and tris(2,4,6-triallyl-1,3,5-triazine) (TAIC) as complementary film-forming precursors. Following chemisorption of PETMP onto the copper surface via Cu–S bond formation, ultraviolet irradiation triggered a thiol–ene click reaction between the pendant thiol groups of PETMP and the allyl functionalities of TAIC, enabling the rapid, room-temperature construction of a densely cross-linked, three-dimensional network film. The resulting film attained a thickness of 77.8 nm and exhibited a corrosion protection efficiency of 99.9% in 3.5 wt% NaCl solution. Li et al. [58] employed pentaerythritol glycidyl ether (PEGE) and 2,5-dimercapto-1,3,4-thiadiazole (DMTD) as complementary film-forming precursors. DMTD was first chemisorbed onto the copper substrate via robust Cu–S bond formation, providing a stable molecular anchor layer. The subsequent optimization of reaction parameters, including temperature, stoichiometric ratio, and curing time, enabled efficient thiol–epoxy click coupling between the anchored DMTD and PEGE, driving progressive, three-dimensional network growth. The resulting hybrid film achieved a thickness of 102 nm and exhibited a corrosion protection efficiency of 99.97% in 3.5 wt% NaCl solution. Notably, after 28-day immersion under identical conditions, the film retained >99% protection efficiency, demonstrating exceptional long-term stability. Li et al. [59] employed water-soluble 3-amino-5-mercapto-1,2,4-triazole (AMT) and glutaraldehyde (GA) as synergistic film-forming precursors. Through sequential interfacial reactions—the initial Cu–S chemisorption of AMT onto the copper surface, followed by Schiff base condensation between the amino groups of surface-bound AMT and the aldehyde functionalities of GA—a robust, cross-linked AMT/GA composite film was formed. This film exhibited an initial corrosion protection efficiency of 99.97% in 3.5 wt% NaCl solution and retained >96% efficiency after seven weeks of immersion, underscoring its remarkable long-term durability. Yang et al. [60] constructed a corrosion-resistant monolayer-derived film on copper via a furan–thiol click reaction between furfuryl mercaptan (FM) and tert-butyl acetylene (TBA). The resulting film achieved a maximum inhibition efficiency of 99.86%, attributable to the formation of stable C–S linkages and the inherent hydrophobicity of the furan–alkyne adduct. Furthermore, strategies integrating surface-selective functional group adsorption, such as amino or carboxyl groups, with orthogonal click chemistries to enable progressive, three-dimensional film growth have been widely explored. Zhang et al. [61] leveraged copper-catalyzed azide–alkyne cycloaddition (CuAAC), utilizing dodecylbenzenesulfonyl azide (DBSA) and 3-butyn-1-ol (BOL) as coupling partners, to fabricate a robust anti-corrosion coating on copper. This CuAAC-derived film delivered a protection efficiency of 96.9%.

#### 3.3.2. Construction of Conversion Coatings Driven by Coordination Effects

When multifunctional molecules serve as film-forming precursors, they undergo strong, site-specific adsorption onto the copper substrate via designated functional groups, such as thiols, amines, or carboxylates. Unreacted pendant functionalities on the anchored molecules then enable dual reaction pathways: (i) covalent cross-linking with complementary reagents to form a robust network, and (ii) coordination with metal ions (e.g., Cu^2+^ or Ni^2+^) present in the solution, wherein the metal ions act as dynamic cross-linking nodes to facilitate continuous, three-dimensional film growth. Chen et al. [64] leveraged the synergistic interaction between L-cysteine and vanadium oxysulfate to construct a hybrid organic–inorganic film on copper via thiol-mediated chemisorption. The resulting coating achieved complete surface coverage and incorporated dispersed vanadium-based oxide nanoparticle aggregates—attributable to coordination bonding between the thiol and carboxylate groups of L-cysteine and vanadium ions in solution. This hybrid architecture exhibited markedly enhanced corrosion resistance compared with conventional single-component organic films. Gongsun et al. [65] addressed dezincification corrosion in brass by co-depositing thioglycolic acid (TGA) and yttrium nitrate to form a dense, self-healing hybrid passivation layer. This film arises from synergistic interfacial processes: (i) thiol-mediated chemisorption of TGA onto copper-rich domains, and (ii) carboxylate–yttrium coordination, wherein Y^3+^ ions which enriched locally due to pH elevation from selective zinc dissolution precipitate as yttrium hydroxide/oxide species. Concurrently, dissolved Cu^2+^ ions coordinate with TGA’s carboxylate groups, enabling dynamic cross-linking and sustained film growth. This dual-metal coordination architecture not only suppresses pitting initiation but also overcomes the kinetic limitation of an insufficient driving force for the continuous monolayer extension of conventional SAMs, thereby delivering robust long-term corrosion protection. Bu et al. [66] constructed a dense organic–inorganic hybrid conversion coating on copper through dual-mode interfacial binding, involving (i) thiol-mediated chemisorption of glutathione onto the substrate and (ii) carboxylate coordination with divalent or trivalent metal ions (specifically Sn^2+^ and Nd^3+^) to induce the in situ formation of metal–organic cross-links. This synergistic architecture delivered a corrosion protection efficiency of 99.89% for copper.

#### 3.3.3. Construction of High-Performance Conversion Coatings Based on Interface Microstructure Regulation

**Figure 6:**
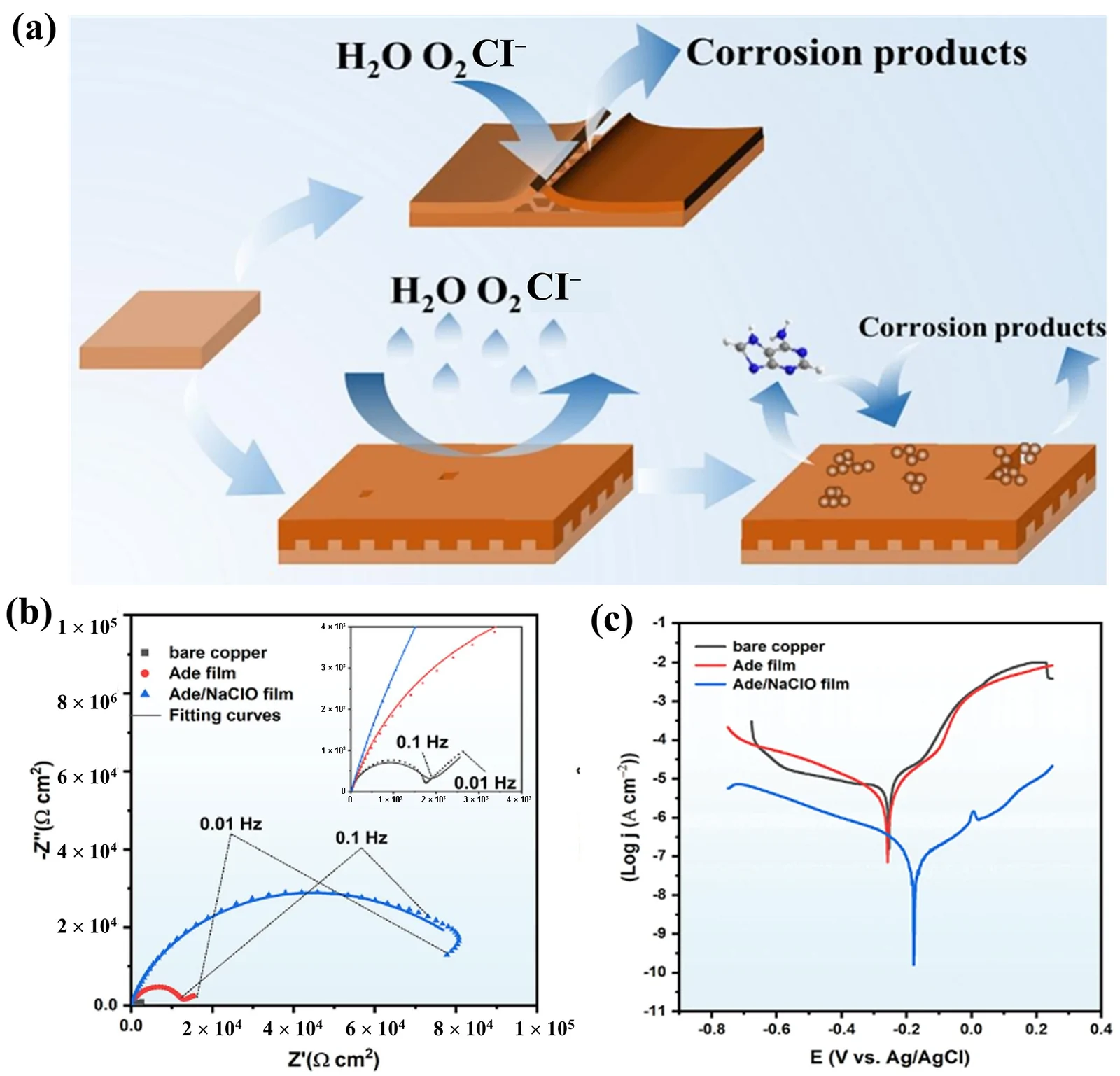
Corrosion protection mechanism (**a**) and anti-corrosion performance (**b**), (**c**) of film with interlocking interface structure [67].

Owing to the inherent chemical inertness of copper, organic films formed solely via characteristic adsorption, such as physisorption or weak chemisorption, often exhibit insufficient interfacial adhesion. This limitation compromises both corrosion inhibition efficacy and long-term structural stability, thereby impeding the industrial adoption of conventional organic conversion coatings. Consequently, rational interfacial engineering to strengthen the copper/film bonding interface, such as through molecular design, co-adsorption, or in situ cross-linking, has emerged as a promising strategy to overcome this fundamental constraint. As shown in Figure 6, Zang et al. [67] employed adenine as a molecular precursor to construct a copper–adenine conversion coating, capitalizing on the strong chemisorption of adenine’s nitrogen atoms, particularly N1 and N3, at copper surface sites. Concurrently, sodium hypochlorite (NaOCl) was applied as a controlled micro-etchant to generate nanoscale surface topography on the copper substrate. This topographical modification promoted preferential adsorption and interfacial confinement of adenine molecules within the etched grooves, yielding a mechanically interlocked copper/film interface that significantly enhanced adhesion strength. Furthermore, Cu^2+^ ions liberated during oxidative etching coordinated with adenine to form stable Cu^2+^–adenine complexes, which infiltrated and healed intrinsic film defects, thereby increasing coating density and structural integrity. The corrosion resistance of films fabricated via this integrated strategy was markedly superior to that of adenine-only reference coatings. Owing to copper’s intrinsic chemical inertness, conventional silane-based SAMs suffer from weak interfacial adhesion and often fail to form uniformly on copper surfaces. To overcome this limitation, Yu et al. [68] engineered a hybrid conversion coating by first anchoring 3-mercaptopropyltrimethoxysilane (PropS-SH) onto copper via robust thiol–copper chemisorption. Subsequently, hydroxyethylidenediphosphonic acid (HEDP) was introduced to reinforce the interface: its phosphonic acid groups (–PO(OH)_2_) coordinate strongly with surface copper sites, while its P–OH moieties concurrently condense with silanol (Si–OH) groups generated during PropS-SH hydrolysis, thereby forming a covalently integrated, defect-suppressed bilayer architecture. The resulting PropS-SH/HEDP composite coating attained a thickness of ~125 nm, exhibited significantly reduced interfacial defects, and delivered a corrosion protection efficiency of 99.95%. After 10 days of immersion in 3.5 wt% NaCl solution, the coating retained a protection efficiency fivefold higher than that of a monolayer silane reference film, demonstrating exceptional long-term stability. Qiu et al. [69] exploited the oxidative capability of molybdate (MoO_4_^2−^) or tungstate (WO_4_^2−^) anions to trigger the growth of a micron-thick conversion coating from water-soluble dithiol 2,3-dimercapto-1-propanol (DOT) on copper. The resulting bilayer architecture comprises (i) an upper polydisulfide layer formed via the oxidative coupling of adsorbed DOT molecules by MoO_4_^2−^/WO_4_^2−^ and (ii) a lower Cu_2_S interfacial layer generated by the reaction between surface-adsorbed thiol groups and Cu^+^ ions released from the substrate. Electrochemical tests indicated that this hierarchical coating achieved a corrosion protection efficiency of 99.0% for pure copper. Conversion coatings engineered via click chemistry or covalent cross-linking exhibit exceptional interfacial bonding strength and film density. However, the main issue with these coatings is the complexity of their multi-step synthesis and the requirement for specific triggers, which may limit their application on large-scale, irregularly shaped copper components. Future research should focus on developing one-pot, catalyst-free synthesis pathways and utilizing bio-based precursors to balance high performance with economic and environmental sustainability.

### 3.4. Recent Advances in Functional Conversion Coatings for Copper Protection

The rapid expansion of strategic emerging industries, including semiconductor chips, integrated circuits, and new energy systems, has subjected copper-based components to increasingly demanding service environments. Operational extremes such as elevated temperatures, high current densities, and high-voltage transients are now commonplace. Conventional conversion coating technologies, however, exhibit insufficient corrosion resistance, thermal stability, and oxidative durability under these conditions, rendering them inadequate for next-generation applications and making innovative research into functional surface protection strategies imperative. Engineering a multifunctional interface on copper substrates that concurrently delivers self-healing capability, exceptional wear resistance, and minimal electrical resistivity will resolve critical technological bottlenecks and enable the sustainable advancement of high-end manufacturing.

#### 3.4.1. Self-Healing Conversion Coatings for Copper

**Figure 7:**
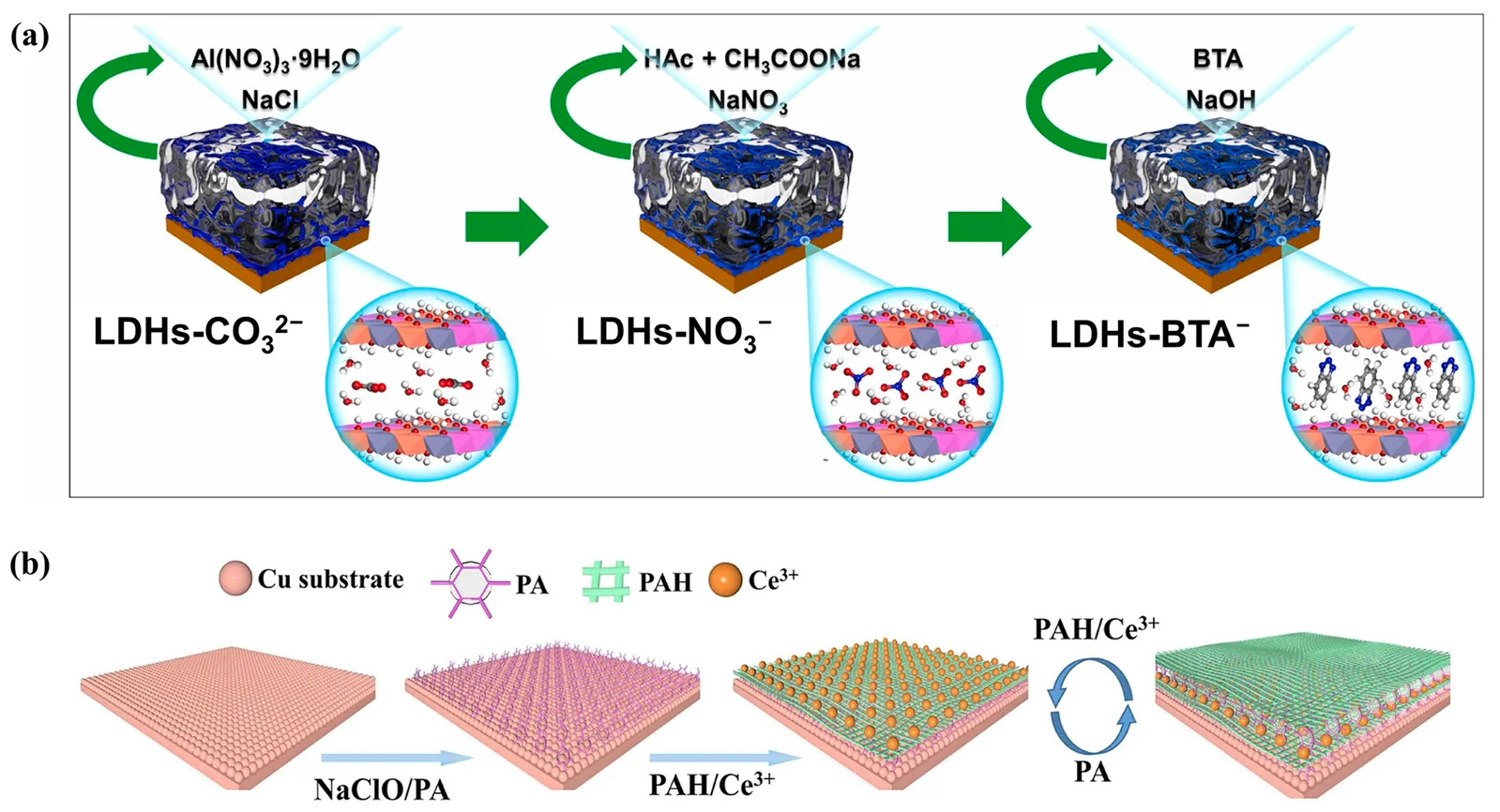
Formation process of self-healing conversion coatings by LDH structure (**a**) [58] and LbL method (**b**) [61].

Self-healing functionality refers to the intrinsic ability of a coating to repair structural damage, including microcracks, mechanical scratches, and interfacial delamination, autonomously or in a stimuli-responsive manner, thereby fully restoring its physical integrity, barrier properties, and electrochemical corrosion resistance. Self-healing conversion coatings significantly extend substrate service life and reduce lifecycle maintenance costs. In contrast to conventional anti-corrosion paints, conversion coatings are inherently ultrathin (typically 1–100 nm) and chemically bonded to the substrate, with attributes that impose stringent constraints on self-healing design. Conventional strategies, including shape-memory polymers or micro/nano-container-based inhibitor release, exhibit limited compatibility with such thin, interfacially anchored architectures due to challenges in dispersion stability, controlled release kinetics, and interfacial adhesion. Consequently, recent efforts to impart a self-healing capability to copper conversion coatings have focused on molecular-level engineering, leveraging dynamic covalent bonds (e.g., disulfides, imines), metal–ligand coordination, or in situ reactive species generation to enable intrinsic, interface-concentrated healing mechanisms. Among these approaches, the in situ construction of layered double hydroxide (LDH) films on copper substrates, followed by the post-synthetic loading of corrosion inhibitors via the materials’ intrinsic anion-exchange capacity or interlayer storage capability, has emerged as a promising strategy to confer self-healing functionality. As shown in Figure 7, Chen et al. [70] achieved in situ growth of a benzotriazole anion (BTA^−^)-intercalated LDH film on brass substrates. Leveraging the intrinsic anion-exchange selectivity of LDHs, they engineered the coating to exhibit active self-healing functionality. Specifically, because the binding energy of Cl^−^ within the LDH interlayer is lower than that of BTA^−^, Cl^−^ ions preferentially exchange into the interlayer upon exposure to chloride-containing corrosive media, thereby releasing the stored BTA^−^ anions to inhibit localized corrosion at defect sites. First, Cl^−^ ions are selectively captured and immobilized within the LDH interlayer, thereby blocking their diffusion pathway to the underlying brass substrate and markedly improving its corrosion resistance. Second, the released BTA^−^ anions coordinate with dissolved Cu^2+^ to form insoluble Cu(II)–BTA complexes at defect sites, enabling the autonomous repair of microscale film damage and establishing a closed-loop “capture–release–repair” protection mechanism. Electrochemical measurements demonstrated that the LDH–BTA composite film achieves an instantaneous corrosion inhibition efficiency of 99.9% and exhibits robust self-healing functionality. Cao et al. [71] fabricated LDH films in situ on copper substrates and concurrently assembled a multilayer architecture by anchoring BTA-loaded electrospun nanofibers onto the LDH surface, thereby integrating dual self-healing mechanisms. Following mechanical damage, the composite film recovered 90.3% of its original corrosion resistance, attributable to the synergistic formation of LDH–BTA adducts and insoluble Cu(I/II)–BTA complexes at defect sites. Matsui et al. [72] fabricated molybdate (MoO_4_^2−^)-intercalated Mg–Al LDH films on Al–Si–Cu alloy substrates, endowing them with both corrosion resistance and active self-healing functionality. Their experimental results revealed that, following 72 h of immersion in a 5 wt% NaCl solution, localized repair was initiated at mechanically damaged sites within the film. Alternatively, the uniform incorporation of corrosion inhibitors via layer-by-layer (LbL) assembly represents a robust strategy for engineering self-healing coatings on copper substrates. Gongsun et al. [73] leveraged the dynamic dissolution–coordination equilibrium of brass in a mixed sodium hypochlorite/PA solution to fabricate a negatively charged copper–phytate pre-deposition layer on the substrate surface. Subsequently, through alternating immersion in a polyallylamine hydrochloride (PAH) solution containing Ce^3+^ ions and a PA solution, they achieved uniform dispersion and spatially controlled deposition of cerium species within the LbL assembly. The in situ-formed CeO_2_ nanoparticles exhibited a high affinity for Cl^−^ adsorption, thereby significantly reinforcing the film’s physical barrier functionality against corrosive electrolytes. Moreover, the reversible Ce(III)/Ce(IV) redox couple enabled passive film regeneration at localized corrosion sites, conferring intrinsic self-healing capability to the coating. Meanwhile, Gongsun et al. [74] also developed a multifunctional protective coating based on octa-thiol polyhedral oligomeric silsesquioxane (POSS–SH), which integrates exceptional wear resistance, hydrophobicity, mechanical flexibility, and autonomous self-healing capability. The coating exhibits a nanohardness of 43.4 MPa, an elastic modulus of 381.0 MPa, a water contact angle of 101.0°, and an elastic recovery ratio of 77.5%, collectively demonstrating outstanding mechanical robustness and deformability. After 30 days of immersion in a 3.5 wt% NaCl solution, it retained a corrosion inhibition efficiency exceeding 99.6%. Critically, the well-defined cage architecture of POSS–SH enables selective halide ion capture, modulates the interfacial electric double-layer structure, and disrupts chloride-induced anodic dissolution, thereby shifting protection from a conventional passive barrier mechanism toward an intelligent “capture–repel–passivate” paradigm. This molecular-level design strategy establishes a new framework for the rational integration of multiple functionalities in high-performance anti-corrosion coatings.

#### 3.4.2. High-Conductivity Conversion Coatings for Copper

In applications such as microelectronics, integrated circuits, semiconductor devices, and emerging energy technologies, a fundamental challenge arises that directly contradicts conventional corrosion protection paradigms: the concurrent realization of exceptional corrosion/oxidation resistance and high electrical conductivity in copper and its alloys under extreme operating conditions. This challenge has transcended traditional single-material protection approaches and has evolved into an interdisciplinary research frontier integrating interface electrochemistry, surface science, and functional materials design [75]. The challenge lies in atomic-scale modulation of the surface electronic structure, which requires not only an effective suppression of environmental degradation but also the preservation or even enhancement of interfacial electron transport efficiency.

**Figure 8:**
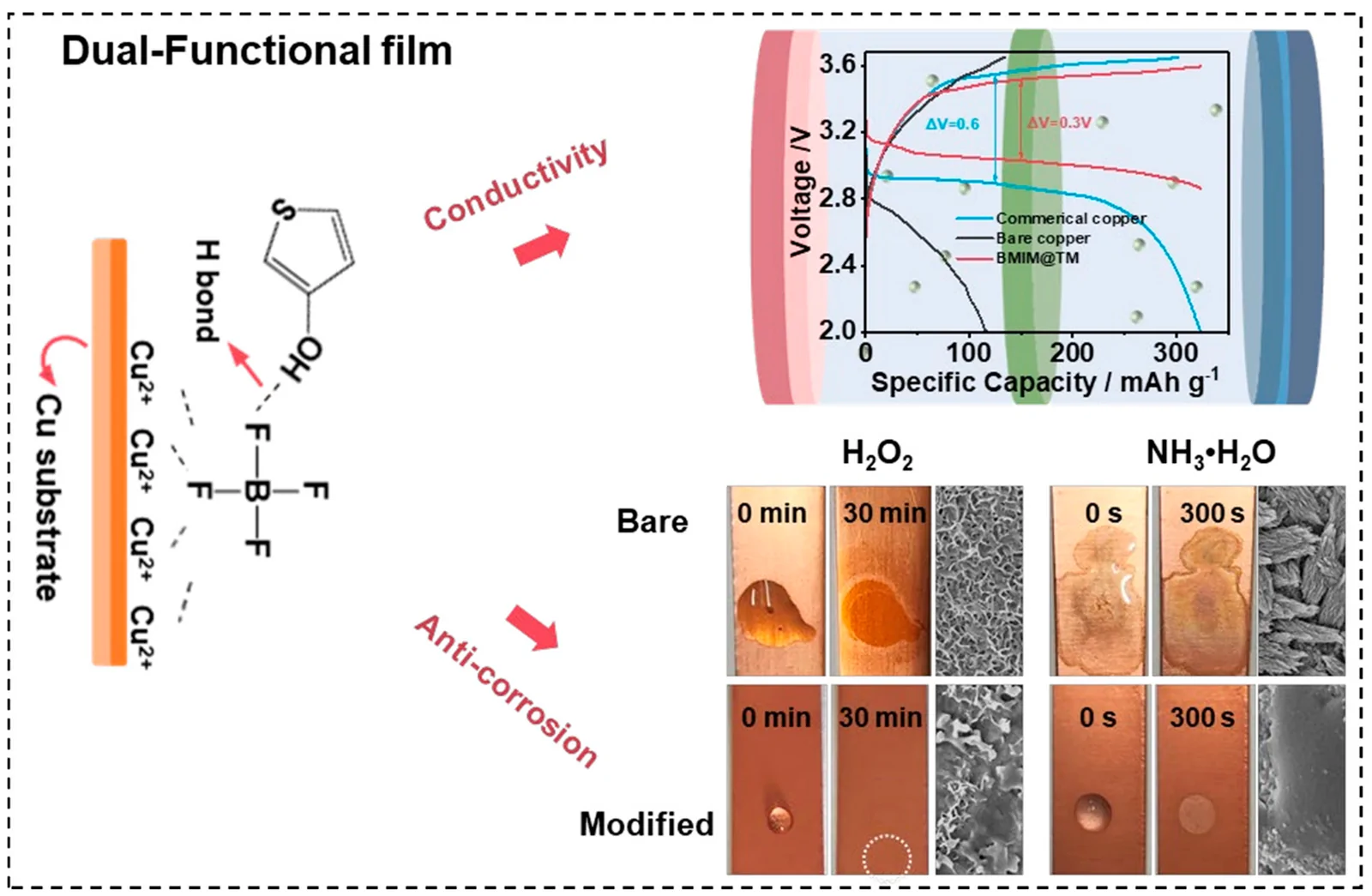
Mechanism and performance of conductive anti-corrosion film-based ionic liquid [65].

The fabrication of conductive polymer coatings on copper substrates is widely regarded as a promising strategy for bipolar plate applications. Li et al. [76] electrochemically synthesized a polyaniline (PANI) film on copper via cyclic voltammetry and systematically evaluated the corrosion resistance and electrical conductivity of bare copper versus PANI-coated copper under simulated proton exchange membrane fuel cell (PEMFC) anode and cathode operating conditions. The PANI coating exhibited a corrosion inhibition efficiency of 92.3% in acidic electrolytes. At a test pressure of 1.5 MPa, the interfacial contact resistance (ICR) of bare copper and PANI-coated copper was only approximately 0.0047 Ω cm^−2^ and 0.0165 Ω cm^−2^, respectively. Furthermore, after an 8-h potentiostatic polarization test at −0.1 V vs. SCE under continuous hydrogen purging, the ICR of PANI-coated copper increased from 0.0165 Ω cm^−2^ to 0.0276 Ω cm^−2^. After an 8-h polarization test at +0.6 V vs. SCE under continuous air purging, the ICR of the PANI-coated copper sample also increased from 0.0165 Ω cm^−2^ to 0.0328 Ω cm^−2^. These results collectively confirmed that the PANI coating simultaneously meets the strict dual requirements of low ICR and high electrochemical stability, thereby validating its suitability for application in copper-based bipolar plates. Yang et al. [77] developed an eco-friendly copper passivator via a scalable, low-cost solution-extraction process using pomelo peel (PP) as a renewable biomass precursor. The resulting passivation layer imparted exceptional corrosion resistance to copper foil in ambient air, chloride-containing aqueous media, and organic electrolytes, without compromising its electrochemical functionality. Notably, when PP-Cu foil was applied as a current collector for lithium-ion batteries, EIS characterization revealed that the semicircle in the medium-to-high frequency region of the PP-Cu foil electrode was substantially smaller than that of the bare copper electrode, indicating a lower interfacial impedance. Rate capability tests demonstrated that the discharge specific capacity of the PP-Cu foil electrode consistently outperformed that of the bare copper foil electrode, and long-term cycling measurements at 1 C further validated this conclusion. Collectively, these results confirmed that modifying the copper surface with a PP coating not only improved the corrosion resistance of the current collector, but also retained its inherent electrical conductivity. As shown in Figure 8, Chen et al. [78] engineered an ultrathin nanocomposite film (~54 nm) on copper substrates via in situ self-assembly, leveraging the synergistic molecular interactions between 1-butyl-3-methylimidazolium tetrafluoroborate ([BMIM][BF_4_]) ionic liquid and 3-thiophenemethanol (TM). Film formation proceeded through a dual-mode stabilization mechanism: (i) the two-dimensional coordination of Cu^2+^ ions with BF_4_^−^ anions, guided by molecular dynamics simulations and characterized by a binding energy of −89.2 kcal/mol, and (ii) π-electron-mediated bridging via the thiophene moiety, which established percolating conductive pathways through electron delocalization. Collectively, this architecture imparted exceptional corrosion resistance and high-temperature oxidation stability to the copper substrate while preserving near-bulk electrical conductivity, thereby resolving the long-standing trade-off between conductivity and protection in conductive metal coatings. Electrical characterization confirmed a surface conductivity of 0.46 MS/cm for the modified copper, corresponding to a 92.3% retention relative to bare copper; EIS and potentiodynamic polarization yielded a corrosion inhibition efficiency of 86.2%, demonstrating a quantitatively balanced resolution of the fundamental performance conflict in functional conductive coatings. Functional coatings incorporating LDH containers or dynamic bonds can provide active protection via autonomous self-healing. While they significantly extend the service life of copper in aggressive media, the balance between the mechanical strength of the matrix and the release kinetics of the healing agents remains a challenge. Over-loading inhibitors can compromise the structural integrity of coatings and barrier effects. Future efforts should prioritize the spatial–temporal control of inhibitor release and the integration of dual-function agents that provide both sensing and healing capabilities. In recent years, industries including new energy, semiconductors, and integrated circuits have been experiencing rapid growth. In these sectors, surface protection for copper and copper alloys not only requires improved corrosion resistance, but also necessitates retaining the intrinsic electrical conductivity of copper. At present, the existing research on enabling surface-protective coatings to simultaneously achieve high corrosion resistance and high electrical conductivity remains relatively limited, and the fundamental design principles for retaining conductivity are still underdeveloped. Accordingly, this research area constitutes a key priority for future scientific and technological investigation.

### 3.5. Critical Discussion and Mechanistic Summary

The transition from corrosion-inhibiting molecules in the laboratory to high-performance coatings is not a random process, but is rather a multi-dimensional evolution involving several key scientific factors. The diverse strategies reviewed—ranging from inorganic conversion to functional hybrids—can be explained through the synergistic effects of micro-scale events and macroscopic electrochemical performance. The thermodynamic foundation starts with molecular adsorption, where the stability of the protective layer is dictated by the energy and properties of the interface bonding. Strong chemical anchoring (e.g., covalent Cu–S bonds or N–Cu coordination) provides the thermodynamic stability required for resisting displacement by aggressive anions, such as Cl^−^. In electrochemical measurements, this is directly reflected in the stabilization of the corrosion potential and the high initial charge transfer resistance. In terms of dynamics, the transition from molecular anchoring to a continuous barrier determines the effectiveness of defect suppression. Whether through chemical adsorption in SAMs or through covalent cross-linking in hybrid networks, film compactness is the primary determinant of barrier integrity. This quality dictates the kinetics of copper dissolution, manifesting as a several-order-of-magnitude reduction in corrosion current density (*i*_corr_). For long-term corrosion resistance, self-healing mechanisms act as a critical line of defense for both pitting and general corrosion. By self-repairing physical crevices or microcracks, these coatings prevent the typical Warburg impedance characteristic of ion diffusion, ensuring that the impedance modulus remains stable even after prolonged immersion in salt spray environments. Moreover, the performance of copper film is highly sensitive to the nature of the corrosive medium. While all reviewed technologies enhance corrosion resistance, their stability profiles vary significantly under pH extremes. The hybrid coatings usually exhibited the highest durability in both acidic and alkaline industrial media. The strong interfacial anchors (such as Cu-S or Cu-Si-O linkages) are more resistant to corrosion of acid and alkali compared to ionically bonded inorganic films, which often dissolve in acidic conditions. Furthermore, the high cross-linking density and inherent hydrophobicity of the hybrid matrix provide a formidable barrier against the penetration of OH^−^ ions, preventing the formation of soluble copper complexes in alkaline environments.

**Figure 9:**
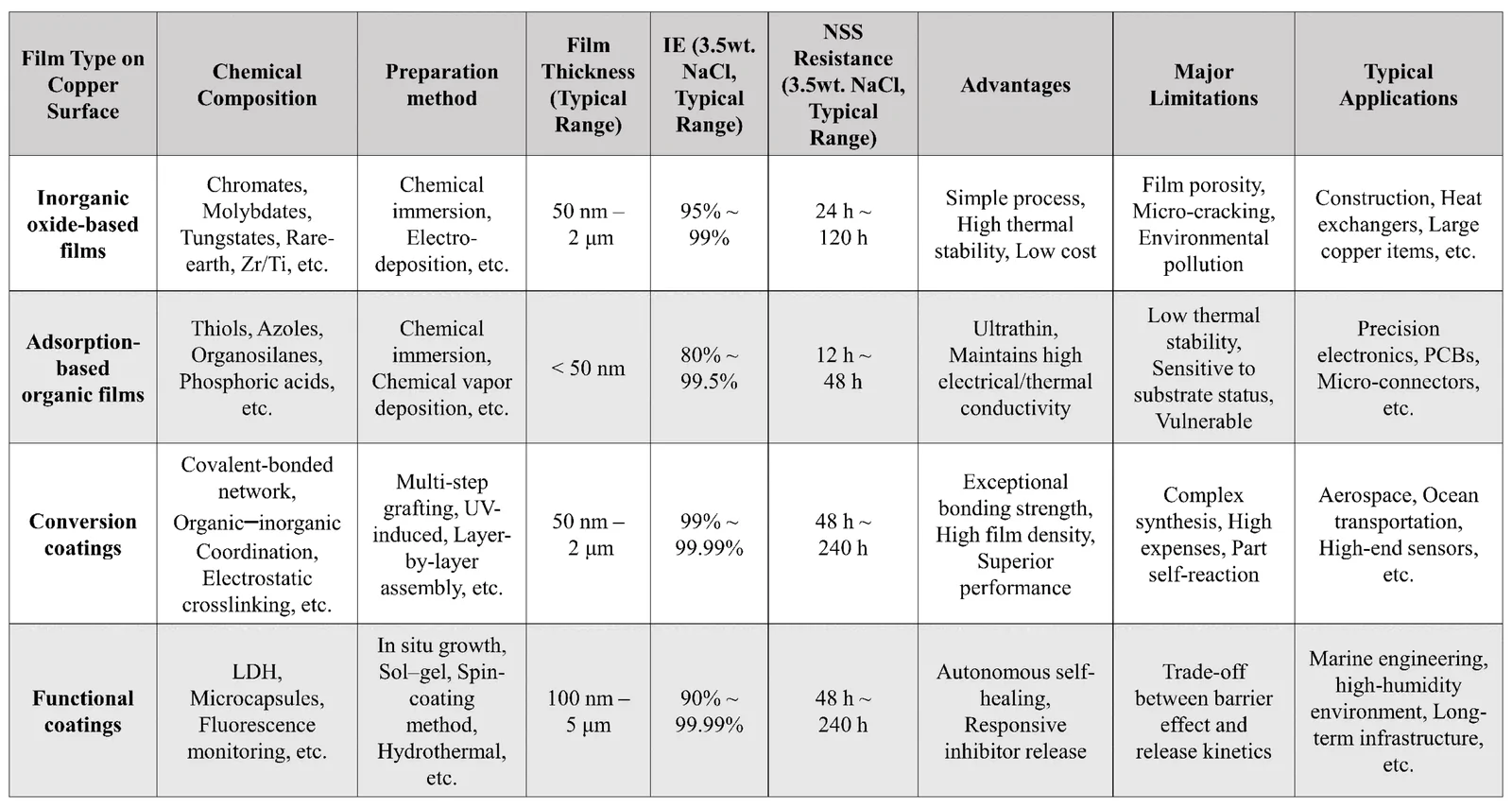
A summary of the types, components, preparation methods, properties, advantages, disadvantages, and applications of the films.

To provide a systematic evaluation of the discussed technologies, Figure 9 compares the four major coating categories based on their chemical composition, preparation methods, and key performance indicators. Inorganic chemical conversion coatings represent the most traditional film technique: when utilizing metal salts such as molybdates and rare earth salts, these coatings typically exhibit a thickness of 50 nm to 2 μm with high thermal stability and low production costs. However, their protection efficiency and salt spray resistance are often constrained by inherent film porosity and microcracking, which remain significant hurdles for long-term industrial applications. Organic self-assembled films provide a stark contrast in terms of scale. With a typical thickness below 50 nm, these films (based on thiols, azoles, or silanes) maintain the critical electrical and thermal conductivity of their copper substrates. Their major limitations lie in their low thermal stability and vulnerability to mechanical wear, resulting in a relatively short lifespan in salt spray environments. This strategy is thus primarily suited for precision electronics and micro-connectors where electrical and thermal conductivity is paramount. Conversion coatings represent a significant increase in performance through molecular-level engineering. By constructing covalent-bonded networks or coordination-driven structures, these coatings achieve exceptional film density and bonding strength. As shown in Figure 9, they exhibit excellent corrosion inhibition efficiencies and significantly extended NSS resistance. The trade-off for such superior performance is the increased complexity of multi-step synthesis and higher operational expenses, making them most applicable for high-performance-requirement sectors such as aerospace engineering and sensors. Lastly, functional and smart coatings introduce dynamic capabilities such as self-healing and fluorescence monitoring. By incorporating LDH containers or microcapsules, these systems can respond to environmental stimuli to release inhibitors. Although these coatings offer long-term corrosion resistance and the ability to repair crevices, the precise control of release kinetics to maintain a balance with the barrier properties of the coating remains a challenge. Overall, this sequential comparison illustrates that, while legacy systems offer simplicity, the field is clearly moving toward advanced, multifunctional hybrid systems that can provide long-term reliability in increasingly aggressive service environments.

In summary, the transition from traditional chromate-based passivation to the environmentally friendly alternatives reviewed herein represents a crucial evolution in copper surface engineering. Historically, chromate systems have been the industrial benchmark due to their robust “active” self-healing capability, which provides exceptional long-term durability in aggressive environments. However, their severe environmental impact, caused by the high toxicity and carcinogenicity of hexavalent chromium, has led to strict global restrictions under RoHS and REACH regulations. Compared to the legacy chromate benchmark, modern eco-friendly coatings—particularly organic–inorganic hybrids and coordination-driven films—offer not only a non-toxic profile but also the distinct advantage of multi-functionality. Nevertheless, the long-term durability of these green alternatives under synergistic multi-physics stressors remains a challenge. Future research must address this gap to ensure that the ecological benefits of chromium-free technologies are matched by the stability required for strategic industrial applications. In addition, with the rapid advancement of strategic emerging industries such as integrated circuits, emerging energy technologies, artificial intelligence, and robotics, copper and its alloys, as foundational functional materials, face increasingly stringent demands for high-temperature oxidation resistance and electrochemical corrosion protection. These challenges extend beyond isolated performance degradation under individual environmental stressors and manifest as coupled-material degradation phenomena driven synergistically by elevated temperature, applied electric fields, mechanical stress, and aggressive chemical species.

## 4. Conclusions and Future Perspectives

Extensive research has advanced chromium-free alternatives—such as inorganic oxidative, organic adsorbed, and chemical conversion films—into viable copper protection strategies. Significant progress has been made in improving the self-healing functionality and electrical conductivity of these films, representing a pivotal evolution toward regulatory-compliant surface engineering. Despite these laboratory achievements, long-term durability under coupled physical stressors remains a critical ongoing challenge. This gap must be bridged to meet the exacting reliability standards of strategic emerging applications, including 3 nm integrated circuits, AI infrastructure, and high-dynamic robotics. In these fields, copper components face multifactorial degradation driven by the synergistic interplay of high temperatures, fluctuating electric fields, mechanical stress, and aggressive corrosive media. Consequently, future developments must aid the transition from a passive physical barrier to multifunctional, adaptive protection systems capable of robust operation within sophisticated multi-physics coupling environments.

(1) In integrated circuit manufacturing, as technology nodes advance to 3 nm, copper interconnects face physical scaling limits, where intensified surface effects and grain-boundary halide migration trigger Kirkendall void formation, necessitating 1–3 nm pinhole-free conformal layers. 

(2) In the energy sector, 800 V EV architectures demand connectors with high arc-ablation resistance, while battery current collectors must suppress lithium dendrites to prevent thermal runaway.

(3) Similarly, high-density AI servers encounter severe Joule heating, which accelerates oxidation and signal insertion loss, highlighting a fundamental tripartite trade-off between electrical conductivity, thermal management, and oxidation resistance.

(4) Furthermore, due to the evolution of robotics, copper conductors are required to endure >106 bending cycles under dynamic loading. Since conventional rigid coatings are prone to microcracking and delamination, next-generation protective films must integrate intrinsic flexibility and biomimetic self-healing to autonomously repair nanoscale damage.

Collectively, these strategic applications transition the research focus from simple passive barriers to multifunctional, adaptive protection systems capable of maintaining reliability within complex, multi-physics environments. Meeting these exacting requirements remains the central bottleneck for next-generation electronic and energy infrastructure.

In conclusion, the central challenge in modern copper protection technology has shifted from achieving passive physical barrier functionality to enabling active, adaptive responses under coupled electrochemical–thermal–mechanical–electrical operating conditions, while simultaneously fulfilling multifunctional requirements. To solve the aforementioned problems and bridge the gap between laboratory research and industrial applications, the following realistic scientific and engineering challenges should be prioritized in the future development of copper films:

(1) Long-term Durability and Environmental Stability: Future studies must evaluate the long-term protective performance and interface integrity of films under complex environments (e.g., concurrent thermal, electrochemical, and mechanical stresses).

(2) Scalability and Cost Considerations: Future efforts should focus on the lab–pilot–industrial scale-up trajectory to ensure the translatability of technology. A rigorous cost assessment is required to balance raw material procurement, transportation logistics, and processing operations, ensuring that the interfacial performance achieved in the lab remains economically sustainable in large-scale production.

(3) Standardized Corrosion Testing: There is an urgent need for standardized evaluation protocols that accurately simulate the specific micro-environments of modern electronics, incorporating in situ monitoring of electrical and thermal conductivity performance during the corrosion process.

(4) Integration with Computational and Machine Learning-Guided Design: Leveraging high-throughput computing and machine learning will be essential to accelerate the rational discovery of synergistic molecular structures and to predict the long-term chemical and structural stability of the film–substrate interface.

## Data Availability

No new data were created or analyzed in this study. Data sharing is not applicable to this article.
